# Multi-omic measurement of mutually exclusive loss-of-function enriches for candidate synthetic lethal gene pairs

**DOI:** 10.1186/s12864-016-2375-1

**Published:** 2016-01-19

**Authors:** Mark Wappett, Austin Dulak, Zheng Rong Yang, Abdullatif Al-Watban, James R. Bradford, Jonathan R. Dry

**Affiliations:** Oncology Innovative Medicines, AstraZeneca, Macclesfield, UK; Oncology Innovative Medicines, AstraZeneca, Waltham, USA; School of Biosciences, University of Exeter, Exeter, UK; Present address: Department of Oncology, University of Sheffield, Sheffield, UK

**Keywords:** Bioinformatics, Transcriptomics, Synthetic lethality, R, Epigenetics, Functional genomics, Cancer, Cell line

## Abstract

**Background:**

Identification of synthetic lethal interactions in cancer cells could offer promising new therapeutic targets. Large-scale functional genomic screening presents an opportunity to test large numbers of cancer synthetic lethal hypotheses. Methods enriching for candidate synthetic lethal targets in molecularly defined cancer cell lines can steer effective design of screening efforts. Loss of one partner of a synthetic lethal gene pair creates a dependency on the other, thus synthetic lethal gene pairs should never show simultaneous loss-of-function. We have developed a computational approach to mine large multi-omic cancer data sets and identify gene pairs with mutually exclusive loss-of-function. Since loss-of-function may not always be genetic, we look for deleterious mutations, gene deletion and/or loss of mRNA expression by bimodality defined with a novel algorithm BiSEp.

**Results:**

Applying this toolkit to both tumour cell line and patient data, we achieve statistically significant enrichment for experimentally validated tumour suppressor genes and synthetic lethal gene pairings. Notably non-reliance on genetic loss reveals a number of known synthetic lethal relationships otherwise missed, resulting in marked improvement over genetic-only predictions. We go on to establish biological rationale surrounding a number of novel candidate synthetic lethal gene pairs with demonstrated dependencies in published cancer cell line shRNA screens.

**Conclusions:**

This work introduces a multi-omic approach to define gene loss-of-function, and enrich for candidate synthetic lethal gene pairs in cell lines testable through functional screens. In doing so, we offer an additional resource to generate new cancer drug target and combination hypotheses. Algorithms discussed are freely available in the BiSEp CRAN package at http://cran.r-project.org/web/packages/BiSEp/index.html.

**Electronic supplementary material:**

The online version of this article (doi:10.1186/s12864-016-2375-1) contains supplementary material, which is available to authorized users.

## Background

Tumour suppressor gene defects drive progression of many cancer types [1, 2], but are poorly served by therapies typically targeting activated oncogenes. Synthetic lethality, defined as a lethal combination of two or more individually non-lethal molecular loss/inhibition-of-function events, offers the potential to exploit tumour suppressor loss therapeutically. Mutations causing loss of function of BRCA1/2 genes, for example, can lead to a deficiency of double strand DNA repair by the homologous recombination pathway and create an exquisite dependency on single strand repair by PARP1/2, resulting in sensitivity to PARP1/2 inhibitors [3, 4]. Functional genomic screening approaches such as CRISPR [5] enable testing of large numbers of synthetic lethal (SL) hypotheses, however methods to enrich for candidate targets linked to inherent molecular deficiencies in cell lines can steer more efficient experimental design. Data driven approaches inferring synthetic lethal gene pairs through mutually exclusive loss-of-function [6] often focus on genetic loss alone using data from a single platform. However loss-of-function may not always manifest at the genetic level for both genes, for example PARP1/2 do not show genetic loss in tumours [7]. Other approaches assume the non-deleted gene will show a reciprocal genetic increase [8], however this is not necessary for manifestation of a dependency. Approaches to uncover mutually exclusive loss-of-function by considering all levels at which loss can be inferred could therefore compliment published approaches and increase the proportion of synthetic lethal pairings detectable in large multi-omic data sets.

Established approaches exist to confidently call genetic loss-of-function from sequencing or copy number data [8, 9]. Defining functionally meaningful ‘low’ mRNA expression, however, can be challenging since the profile for many genes shows a normal distribution [10] and tumour samples typically comprise a heterogeneous mixture of cell types. Decreases in gene expression resulting from a lack of transcription factor activation, changes in cell state [11], complete or partial gene deletion, or point mutation of a tumour suppressor gene, can result in loss of a detectable mRNA product [12]. Outlier detection methods are regularly applied to detect extreme increases in expression caused by gene fusion events with highly active transcription factors (ETV1-TMPRSS2 in prostate cancer, [13]) or gene amplifications (ERBB2 in breast cancer), but loss events can appear more subtly or be masked by the background noise expected from ‘omic platforms. PARP1, for example, does not show genetic loss in tumours but does display a markedly low mRNA level in a number of patient tumor samples [7]. Bimodality and non-normality offer a route by which we may identify more subtle changes in gene expression state that remain prominent enough to clearly stratify and classify patient samples [10], and thereby a useful tool to infer loss-of-function through loss of expression.

**Fig. 1:**
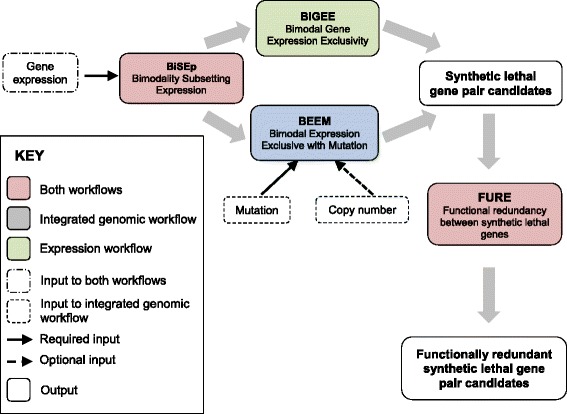
Synthetic lethality toolkit and workflow. Each of the BiSEp, BiGEE, BEEM and FuRE modules are specific functions in the R package. The object generated by BiSEp is the input to the BiGEE and BEEM tools. Additional plotting functions are available for each of the BiGEE and BEEM modules. The expression workflow is run using the tools BiSEp then BiGEE then FuRE. The genomic workflow is run using the tools BiSEp then BEEM then FuRE

We have developed a workflow to enrich for candidate synthetic lethalities from multi-omic data for testing in loss-of-function screens (Fig. 1). We present this workflow as a set of computational tools which enable the user to detect gene-gene pairings with mutually exclusive loss-of-function defined by pre-annotated deleterious mutation or gene deletion, and/or loss of mRNA expression. To more comprehensively identify loss of mRNA expression, we introduce a novel algorithm BiSEp (Bimodality Subsetting Expression) to partition low from high mRNA expression where visible as bimodality or non-normality. We further enrich for candidate synthetic lethality by filtering for gene pairs with biological functional redundancy, inferring one may compensate for the other. We have applied this workflow to publicly available data sets for large panels of tumour cell lines [14, 15] and tumour patient datasets published by the TCGA consortium [16]. Comparing to established methodologies [8, 9] we show statistical enrichment between gene pairs found, but also uniquely identify candidate SL gene pairs of biological relevance. We demonstrate accurate re-identification of known synthetic lethal targets in cancer; nominate several novel candidate synthetic lethal interactions involving at least one tumour suppressor; show statistical enrichment of gene pairs with experimental evidence of lethality in yeast screens. Six interactions are further supported by in vitro data from a large RNAi screen in human cancer cell lines, and their biological rationale is discussed.

## Methods

Algorithms described below are available as individual functions available in the R toolkit BiSEp. For further instruction and examples, please see the vignette at http://cran.r-project.org/web/packages/BiSEp/index.html.

### Cell line and tumour patient data preparation

Affymetrix HG U133 plus 2 microarray gene expression data for 811 cancer cell lines from the Cancer Cell Line Encyclopedia (CCLE) dataset were downloaded from the Gene Expression Omnibus (GSE36133). Analyses were performed on cell lines derived from solid tumours only. Multi-targeting probesets were removed and normalization performed using the frozen RMA approach [17]. Resulting probe-level signals were summarized to the gene level in linear form and scaled between 1 and 100 across cell lines prior to transformation back to the log2 scale.

Exome sequencing mutation data from 442 Sanger cell lines matched to a cell line from the CCLE was downloaded from COSMIC [15]. Genes with at least one heterozygous or homozygous mutation were classed as mutated and assigned a “MUT” call. All other genes were classed as wild type (“WT”), and then the results formatted as a gene-by-sample matrix. The functional consequence of all mutations is considered by default to be loss, other than for silent mutation calls which were removed. Gene-level copy number data for the same cell lines were downloaded from the CCLE and summarized to a “loss” or “normal” call where loss was defined as <1 copy assuming the majority of cell lines are diploid. These data were then combined with the mutation data to generate a mutation/copy number matrix in which a gene with a mutation, and/or evidence of copy number loss is assigned a “MUT” call and “WT” otherwise.

RNA-Seqv2 gene-level expression data for 178 lung adenocarcinoma tumour patient samples were downloaded from TCGA and RSEM [18] normalized expression values were formatted to a gene-by-sample matrix. TCGA exome sequencing mutation data for the same samples were also downloaded from the Firehose resource using the MAF dashboard (January 2014). The variants were pre-computed and summarised as above.

### Defining expression loss with BiSEp (Bimodal Subsetting of Expression)

The BiSEp tool is comprised of two separate components - the published Bimodality Index algorithm [10] and a novel algorithm called BIG.

#### The Bimodality Index (BI) [10]

Given a gene expression profile across a population of samples, the Bimodality Index algorithm attempts to detect the mixture of two normally distributed populations. It reports back the mean expression level of each of the two populations, the distribution of samples across the two populations (π), the distance between the two populations (δ) and an overall measure of bimodality (BI). The larger the values of BI, the easier the bimodal populations are to distinguish from each other [10].

#### Bimodality In Genomics (BIG)

The focus of BIG is to accurately detect the midpoint of the bimodal distributed genes, including those whose bimodality may be diluted (as likely in a heterogeneous cell population) to a more non-normal profile. In order to do this we first sort expressions of each gene. Based on the sorted expressions, we then calculated the distance between every pair of consecutive sorted expressions. This process generated a vector of distances between sorted expressions, which was again sorted leading to a working vector (see Additional file 1 for full methodology). The point of maximum distance leads to the mid-point call for each bimodal / non-normal gene.

The BISEP tool therefore combines BI, π and δ values with the distribution midpoint value derived by BIG to detect both bimodal and non-normal expression profiles. This provides the user with more control over the shape of the bimodal distribution, and enables the dynamic partitioning of samples into high and low populations.

### Determining thresholds separating high vs. low bimodal expression

Four default parameter settings are available, ‘cell_line’, ‘cell_line_low’, ‘patient’, ‘patient_low’, that change the bimodal expression filters (BI score and δ) to reflect the differences between homogeneous cell lines and more heterogeneous patient populations, as well as sample power. Based on these prior assumptions, ‘patient’ parameters result in less stringent bimodality filters to account for dilution of bimodal signals (and resulting overlap of modes or non-normality) expected in heterogeneous samples, and “_low” parameters should be applied to datasets with fewer than 200 samples in order to reduce the false positive rate.

**Table:** 

Parameter	Filters
cell_line:	BI = 0.7, δ = 2.5
cell_line_low:	BI = 1.1, δ = 3.5
patient:	BI = 0.5, δ = 2.5
patient_low:	BI = 0.9, δ = 3.0

The defaults are a guide and may be changed at the user’s discretion.

For any bimodal gene determined by BiSEp, the midpoint separating the two modal distributions (see Additional file 1) is used to partition the samples into “low” and “high” expressing classes. For most bimodal genes the two ‘modes’ overlap (Additional file 1: Figure S2), therefore the tool allows a window of up to 5 % of the distance from the mid-point to the bottom of the dynamic range in which samples are not assigned a definitive high/low expression classification. This window varies based on the balance of the bimodal distribution – if there are fewer samples with a ‘low’ classification and higher likelihood of mode overlap (‘patient’ like) then the window size will be closer to 5 %, whereas if it is more balanced with clearer separation between the two modes (‘cell_line’ like) then the window will tend towards 0 %.

### Mutually Exclusive Mutations (MEMU)

To identify synthetic lethalities visible as genetic loss alone, all possible gene pairs from the Sanger exome sequencing project [15] were evaluated for evidence of mutually exclusive somatic mutation or gene deletion. Pairs of genes were evaluated for mutual exclusivity using a two-tailed fisher exact test, and only genes with a mutation rate of greater than or equal to 5 % were considered. An odds ratio of < 2.5 and *p* value of < 0.05 (FDR of < 0.1) were used to define the mutually exclusive population size.

### Bimodal-low Gene Expression Exclusivity (BiGEE)

BiGEE takes the output from BiSEp in the form of a log_2_ expression data matrix of genes identified as significantly bimodal. BiGEE iteratively assesses all pairwise combinations of bimodal expression profiles for mutually exclusive low expression as evidence of potential synthetic lethality. For any gene pair, the midpoints of the two bimodal distributions (see Additional file 1) are used to partition the data into four quadrants separating samples with high and low expression of each gene respectively. Gene pairs with less than 1 % of samples classified ‘low’ for both genes around the adjusted midpoints are classed as potentially synthetic lethal. The remaining gene pairs are scored for synthetic lethality using a combination of the δ (distance between two expression modes), π (proportion of samples in each expression mode) and BI variables calculated when detecting bimodality using the following equation where x and y are the members of the gene pair:

$$ S={\displaystyle \sum }x\left(\pi \right),\ y\left(\pi \right),\ {\displaystyle \sum }x(BI),\ y(BI),\ {\displaystyle \sum }x\left(\delta \right),\ y\left(\delta \right) $$

Gene pairs that contain genes with greater distance between the high/low populations, and a more even balance in sample numbers between the two populations will score more highly using this metric (Additional file 1: Figure S2) although all gene pairs with a mutually exclusive low signature are returned. The tool returns a matrix containing gene pairs ranked according to S score, although the primary measure of significance is mutual exclusivity (all returned pairs).

### Bimodal Expression Exclusive with Mutation (BEEM)

Gene mutations may result in loss of gene function without loss of mRNA expression. We developed an algorithm, BEEM, to evaluate all bimodal expression profiles of genes for their relationship with all gene mutation profiles. BEEM takes as input the output of BiSEp, and a mutation data matrix of discreet ‘WT’ or ‘MUT’ mutation calls where rows are genes and columns are samples. This may be based on presence or absence of somatic mutation calls typically generated by data from exome sequencing experiments [19], or a discreet call for a gene level copy number deletion. As such, the most appropriate datasets for use with BEEM should consist of expression and mutation data types matched at the sample level. The tool utilises the output from the BiSEp method to identify bimodal expressed genes in the expression matrix and pre-filters the mutation matrix based on a population-based frequency filter defined by the user. Prevalence of mutation in each of the remaining genes is assessed in both the low and high expression modes of the bimodally expressed genes. Significance of enrichment is evaluated for mutation status in the high expressing populations by iteratively populating a contingency table and performing a Fisher’s exact test. To find candidate synthetic lethal gene pairs, mutations that are enriched or entirely mutually exclusive (*p* < 0.25) where the gene expression population is high (and exclusive to the low expression population) are prioritized. This liberal threshold of *p* < 0.25 is implemented in order to maximize inclusion of mutually exclusive gene pairs where the expression gene has a small low expression mode. Output consists of a list of gene pairs ranked according to significance (*p*-value), with accompanying mutation frequency for the low and high expression modes.

### Functional REdundancy between synthetic lethal genes (FuRE)

Assuming each gene in a truly synthetic lethal pair needs to functionally compensate for the other, we aimed to prioritize functionally redundant gene pairs. FuRE accepts user-defined ranked lists of potentially synthetic lethal gene pairs in the format output from either MEMU, BiGEE or BEEM. These gene pairs are annotated with Gene Ontology (GO) terms using a combination of the GO database Bioconductor package GO.db [20] and the Homo sapiens annotation Bioconductor package org.Hs.eg.db [21], and their functional similarity calculated using the Bioconductor package GOSemSim [22]. This package enables the retrieval of all gene ontology information associated with a gene pair, and highlights any meaningful relationships based on this. Each gene pair is assigned a semantic similarity score between 0 and 1, with higher scores indicating a greater similarity. Output consists of a list of gene pairs ranked by synthetic lethality potential from BiGEE/BEEM marked-up with semantic similarity score.

### Evaluation of output from synthetic lethality enrichment workflows

#### Yeast synthetic-lethal-screen validation

As relatively few human synthetic lethal pairs have been confirmed and released into the public domain, we used a set of 24,407 synthetic sick/lethal interactions found in yeast [23, 24] as a surrogate for an equivalent human set. Yeast gene pairs were mapped to their human orthologues resulting in a final set of 121,194 human gene pairs referred to as the ‘yeast SL set’. These gene pairs were then overlapped with outputs from the MEMU/BiGEE/BEEM/FuRE workflows where at least one gene from the SL candidate pairs mapped to the yeast SL set. Significance was calculated using a random permutation test in which x gene pairs were drawn at random 10000 times from all possible gene pairs in the human genome where x is equivalent to the number of gene pairs predicted as synthetic lethal by the outputs from the MEMU, BiGEE or BEEM workflows.

#### Human synthetic-lethal screen validation

Two recent publications have identified sets of candidate synthetic lethal interactions from large-scale cancer genomics data using complementary methods [8, 9]. Lu et al. [9] report > 590,000 candidate synthetic lethal interactions that we compare to the BiGEE/BEEM/FuRE workflow ouput using the same permutation method described above. The “DAISY” approach reported by Jerby-Arnon et al. [8] reports a more concise list of 2,816 candidate synthetic lethal pairs and 3,635 synthetic-dosage lethal pairs. Here we have looked for enrichment of bimodal genes featuring in our gene pairs in the DAISY synthetic lethal and synthetic-dosage lethal gene lists using a fisher exact test.

#### RNAi *in silico* validation

We use epigenetic RNAi screen data from human cancer cell lines to further assess the validity of the predictions made by the MEMU/BiGEE/BEEM/FuRE workflows [25, 26]. ATARIS solutions were generated by running the ATARIS algorithm [25] on normalized and aggregated epigenetic RNAi screening data with default parameters. Data reports provide the relative dependency, or phenotype score, of a cell line for a given gene silencing in the context of a second gene mutation. The Hoffman epigenetic screen included the analysis of 260 genes, and 57 cell lines that overlapped with CCLE. This overlap supplied enough statistical power to test many of the epigenetic gene pairs nominated by our toolkit. Where at least one gene from the predicted SL pair matched a gene from RNAi, we looked for evidence of significant correlation between mutation, copy number or expression loss and, Hoffman screen phenotype score using a Wilcoxon rank sum test and a *p* < 0.05. In other words where there is integrated-genomic loss of one gene, there is greater dependency upon knock-down of the partner derived using our workflows – adding confidence to the gene pairs as candidate synthetic lethal. The script used to perform RNAi validation is provided as Additional file 2.

#### Functional prioritization

Following the precedent for synthetic lethality in DNA repair and metabolic signaling, and the clinical opportunity for synthetic lethal partners to tumor suppressor genes, we sought to prioritize respective output from the BiGEE/BEEM/FuRE workflows. We mapped to all tumour suppressor genes [27], all known DNA repair genes (GO:0006281), and finally all cell metabolism genes [28]. A Fisher’s exact test was used to measure the enrichment of these functional gene lists in the output from the pipelines.

## Results

### Overview of synthetic lethality enrichment workflows

We hypothesized that synthetic lethal gene pairs, where loss of one gene creates an exquisite dependency on the other, will show functional redundancy and mutual exclusivity of loss-of-function characteristics. We further hypothesized that relevant loss-of-function may not always be determined by a genetic event, and that gene mutation may cause loss-of-function without loss of an mRNA product. Finally we hypothesized that loss-of-function may be inferred by low mRNA expression where clearly differentiated through bimodality or non-normality with the algorithm BiSEp. To enrich for candidate synthetic lethal gene pairs, data-driven workflows were created to identify mutually exclusive loss-of-function defined as either deleterious mutation, copy number loss, and/or low mRNA expression:

1. Genetic-only workflow: searches for mutual exclusivity of genetic (mutation and/or copy number) loss using a combination of MEMU and FuRE.
2. Integrated-genomic workflow: searches for loss of expression, mutually exclusive to mutation and/or copy number loss using a combination of BiSEp, BEEM and FuRE.
3. Expression-only workflow: uses exclusively gene expression data searches for mutually exclusive loss of expression through a combination of BiSEp, BiGEE and FuRE.

A schematic illustrating these workflows is shown in Fig. 1.

### BiSEp is sensitive to delineating expression in both homogenous and heterogeneous cell populations

**Fig. 2:**
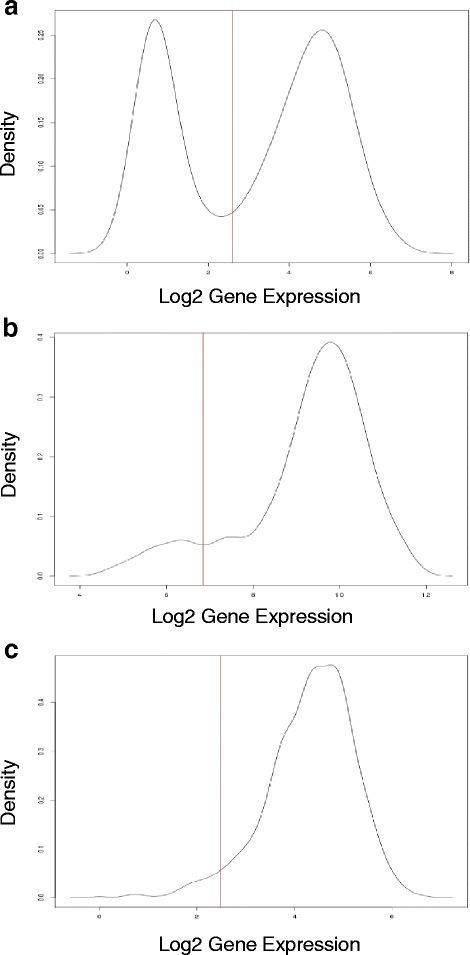
Detecting genes with different expression modalities. **a** The log2 expression density distribution of LDOC1 across 811 CCLE cell lines. LDOC1 has a strong on/off state typical of tumour suppressor genes in homogeneous cell populations. Additionally LDOC1 has been linked as a regulator to NF-KB which in turn promotes the epithelial-mesenchymal transition. **b** The log2 expression density distribution of LDOC1 across 101 TCGA lung adenocarcinoma patients. LDOC1 has a diluted, non-normal profile - a reflection of the heterogeneity of the tumour tissue. BiSEp can still detect this diluted profile in tumour data. **c** The log2 expression density distribution of PARP3 across 811 CCLE cell lines

Using LDOC1 and PARP3 as examples, we tested the sensitivity of BiSEp in detecting the mid-point of distributions from two populations with different degrees of bimodality. LDOC1 (leucine-zipper down-regulated in cancer) is a tumour suppressor gene that is expressed in most normal tissue, but lost in some cancers [29]. LDOC1 is involved in negative regulation of cell proliferation through the transcription factor NF-KB (critical to the epithelial - mesenchymal transition) and is often lost in cell lines [29]. In the homogeneous cell line dataset from the CCLE [14], LDOC1 displays a classic bimodal distribution with two almost equally distributed populations (Fig. 2a). In the more heterogeneous lung adenocarcinoma dataset from TCGA, the bimodality is not as clear, presenting as a non-normal distribution (Fig. 2b) and reflecting the effect of mixed clonality on tumour expression datasets. Nevertheless, BiSEp is still able to correctly detect the boundary between the two populations in the patient data. As a second example, it is important that BiSEp is capable of detecting genes like PARP3 with non-normal distributions and a small population within the low expressing group, a likely profile of genes in synthetic lethal relationships. BiSEp nominates a midpoint of the non-normal distribution of PARP3 cell line expression data, even when non-obvious (Fig. 2c). These results not only highlight the importance of in vitro homogeneous cell populations in modeling specific cancer cell states, but also the sensitivity of the BiSEp approach in delineating both homogenous and heterogeneous sample populations and addressing the challenges of identifying bimodal gene expression distributions driven by only one or a few cell types.

### BiSEp bimodal-low expression correctly enriches for tumour suppressor genes

Synthetic lethal pairings to known tumor suppressors are of particular importance as they may present the opportunity to prosecute a tumour suppressor population (as in the case of BRCA / PARP). Since multiple tumor suppressors are lost through gene deletion, one would expect an enriched detection when looking for loss of mRNA expression, thus both the expression-only and integrated-genomic workflows were of interest. Analysis of the cell line expression-only workflow results revealed 194 gene pairs where both members are a confirmed tumour suppressor gene and a further 8,777 where at least one of the members is a confirmed tumour suppressor gene (and matches the BiSEp bimodality criteria). Analysis of the cell line integrated-genomic workflow results revealed 413 gene pairs where both members are a confirmed tumour suppressor gene and a further 11,090 where at least one of the members is a confirmed tumour suppressor gene. We assessed the enrichment of tumour suppressor pairs in the outcomes of the expression-only and integrated-genomic workflows by comparing the number of tumour suppressors within gene pairs with those not in gene pairs. The enrichment *p* value for the expression-only workflow is < 1 × 10–9, and for the genomic workflow is < 0.008 (Additional file 1: Figure S3c). To test the ability of the toolkit to detect tumour suppressor genes in tumour data we performed a similar comparison on the outputs of expression-only and integrated-genomic workflow runs in the TCGA lung adenocarcinoma dataset (Additional file 1: Figure S3d, Additional file 3: Tables S8 and S9). The enrichment *p* value for the lung adenocarcinoma genomic analysis is < 0.007. For the expression analysis, although there is a higher percentage of tumour suppressors within the gene pairs it does not reach statistical significance (*p* value < 0.21). The enrichment of tumor suppressor genes identified with bimodal-low expression by BiSEp corroborates the relevance of this approach to detect meaningful loss-of-function.

### Comparison of workflows to enrich for synthetic lethality in yeast screens

**Fig. 3:**
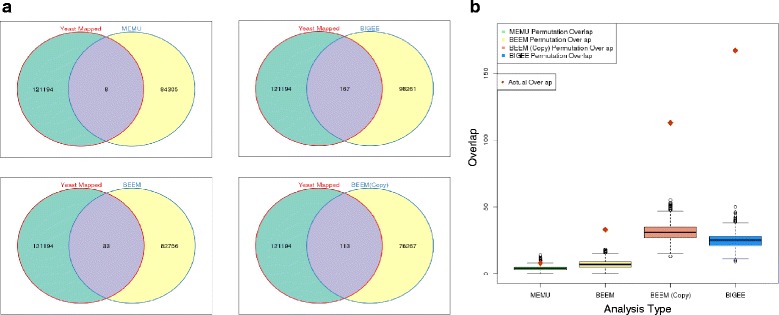
Evaluating toolkit performance with permutation analysis. **a**The gene pair outputs of mutually exclusive mutation analysis (MEMU), the expression workflow (BiGEE), and the genomic workflow (BEEM) (with and without discreet copy number calls), are overlapped with the human orthologues of synthetic lethal yeast gene pairs. **b** In each case, this intersect is compared to 10,000 random samplings of the same size of the gene pair outputs to establish how the workflows perform when compared to chance

A typical approach to predicting SL pairs is to identify mutually exclusively mutated gene pairs. For all individual genes with mutations in greater than or equal to 5 % of samples from the Sanger exome sequencing project [15] we performed a genetic-only workflow, evaluating all possible gene pairs achieving a for evidence of mutually exclusive somatic mutation using a two-tailed Fisher exact test. Gene pairs achieving an odds ratio >2.5 and *p* < 0.05 were classed as mutually exclusive, enabling capture of candidate mutual exclusivity in a population where most gene pairs are double wild-type. 84,305 gene pairs met the threshold, only 8 of which were classed as SL in the Costanzo [23] yeast synthetic lethal screen (*p* = 0.07; Fig. 3, Additional file 1: Figure S4).

We next applied an integrated-genomic workflow using mRNA expression and mutation data across 442 cell lines and revealed 82,756 gene pairs with enrichment or exclusivity of the mutant gene in samples defined as highly expressed by BiSEp. Comparison with the Yeast SL set resulted in a significant overlap of 33 gene pairs (*p* < 0.0001; Fig. 3; Additional file 3: Table S1). Running the same workflow using both copy number loss and mutation to classify a gene as “MUT” revealed 76,267 gene pairs, 113 of which overlapped with the Yeast SL set (*p* < 0.0001, Fig. 3, Additional file 3: Table S3).

Finally we applied an expression-only workflow across 811 cell lines to identify 98,261 gene pairs that are never expressed at low levels (as determined by BiSEp) together, and demonstrating significant overlap with the Yeast SL set (167 gene pairs, *p* < 0.0001; Fig. 3; Additional file 3: Table S2). Taken together, these results strongly suggest that the reduction in search space by ~99.9 % from ~380 million gene pairs to <100,000 by either of these approaches leads to an improved enrichment of true synthetic lethal pairs, and that inclusion of loss-of-function inferred by bimodal-low mRNA expression further improves this enrichment complimenting the more typical approach of identifying mutually exclusive genetic loss only. When prioritized gene pairs are partitioned into target genes aligned to cell lines harboring the paired molecular loss, these data present reasonable numbers for further hypothesis testing in rationally designed siRNA or CRISPR loss-of-function screens

### Comparison of workflows to enrich for synthetic lethality in Human screens

First we compared the 82,756 gene pair output from the integrated-genomic workflow and performed a comparison with the long-list output from Lu et al. [9]. A significant overlap of 76 gene pairs (*p* < 0.018; Additional file 1: Figure S5; Additional file 3: Table S7) was seen when compared to 10,000 random samplings of gene pairs. Similarly the 98,261 gene pair output from the expression-only workflow showed a significant overlap of 420 gene pairs (*p* < 0.0001; Additional file 1: Figure S5; Additional file 3: Table S6) to the long-list from Lu et al. [9] when compared to 10,000 random gene pair samplings.

Little gene pair overlap was seen to results reported by Jerby-Arnon et al. To test similarity of individual gene coverage amongst candidate synthetic lethal gene pairs, we examined enrichment between the 1,007 genes found in the 98,261 gene pairs reported by the expression-only workflow to genes reported in the Jerby-Arnon et al. synthetic lethal (SL) and synthetic-dosage lethal (SDL) genesets (Additional file 1: Figure S5). A significant enrichment is seen to both the SL (*p* < 1.08E-25) and the SDL genesets (*p* < 7.03E-16). Finally we examined the enrichment of the 1,268 genes found in the 82,756 integrated-genomic workflow gene pairs, similarly finding a significant enrichment of the integrated genomic workflow gene pairs in both the Jerby-Arnon et al. SL (*p* < 1.09E-42) and SDL (*p* < 7.03E-35) genesets.

### Analysis workflow output validates existing DNA repair gene pairs and identifies a compelling new pair: ERCC4 and XRCC1

**Fig. 4:**
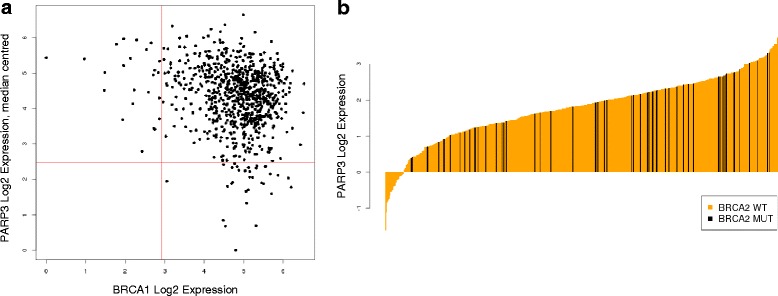
BRCA and PARP synthetic lethality is identified by the BiSEp toolkit. **a** The expression distribution of BRCA1 and PARP3 across 811 CCLE cell lines. This pair was detected by BiGEE as the two genes are never expressed at low levels together (below the bimodal midpoint of expression). **b** The expression distribution of PARP3 (orange) coloured by BRCA2 loss (black). This pair was detected by the BEEM analysis. BRCA2 mutation is never seen where PARP3 expression is low

To further add confidence to the approach outlined above, included in the outputs were well-established synthetic lethality relationships between the homologous recombination protein BRCA and the base excision repair PARP protein family [3]. This was visible as a mutually exclusive loss of gene expression between BRCA1 and PARP3 using the expression-only workflow (Fig. 4a). The integrated-genomic workflow also indicated that BRCA2 is wild type in samples with low levels of PARP3 expression suggesting both of these genes cannot be lost simultaneously and inferring that a synthetic lethality relationship exists (Fig. 4b).

We next studied the outputs of the integrated-genomic and expression-only workflows for enrichment of DNA Repair genes using the using the term GO: 0006281 (Additional file 1: Figure S3a). Analysis of the expression-alone workflow reveals 22 DNA repair genes overlap with the output (*p* = 0.059). Analysis of the genomic workflow reveals an overlap of 33 DNA repair genes with the output (*p* = 0.21).

The most compelling gene pair involving two DNA repair genes was ERCC4 and XRCC1 (S = 0.24, Additional file 1: Figure S3b). XRCC1 is a recognized tumour suppressor gene lost in breast cancer. It is integral to single-strand DNA repair by the base excision repair pathway. ERCC4 is a member of the nucleotide excision repair pathway that complexes with ERCC1 and is involved in the removal of platinum adducts. ERCC4 has been shown to have a key role in the initiation of double-strand break repair, caused by stalled replication [30] common in XRCC1 deficient cells [31]. We postulate that loss of single-strand DNA repair capacity through XRCC1 deletion in breast cancers creates a dependency on nucleotide excision repair by ERCC4.

### In silico validation of nominated pairs using publically-available epigenetic shRNA screen data: expression-only workflow

Inhibiting various components of the epigenetic machinery can restore chromatin function affected by abnormalities in epigenetic genes in cancer [26]. Therefore, there is significant interest in identification of new therapeutic targets with a synthetic lethal hypothesis associated with epigenetics. To explore the potential of the BiSEp toolkit to generate epigenetic-relevant synthetic lethal candidates, output from the expression-only workflow were overlapped with the 260 genes from the Hoffman epigenetic screen. There were 5,213 gene pairs that included at least one epigenetic gene and 54 pairs where both members were epigenetic genes (Additional file 3: Table S5). These outputs were combined with the RNAi dependency data from the Hoffman yeast screen to identify the most compelling candidate synthetic lethal targets. Four gene pairs were investigated in further detail and are described below.

#### SMARCA1 and SMARCA4

**Fig. 5:**
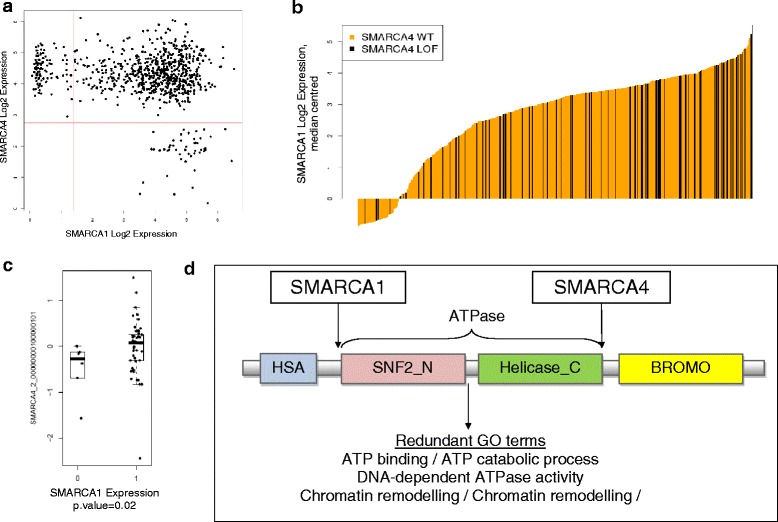
The SMARCA4 and SMARCA1 synthetic lethal pairing. **a** The expression distribution of SMARCA4 and SMARCA1 across 811 CCLE cell lines. This pair was detected by BiGEE as the two genes are never expressed at low levels together (below the bimodal midpoint of expression). **b** The expression distribution of SMARCA1 (orange) coloured by SMARCA4 loss (black). This pair was also detected by the BEEM tool (with copy number data). SMARCA4 loss is enriched where SMARCA1 expression is high. This is indicative of possible interdependency. **c** The ATARIS phenotype dependency scores for cell lines on the SMARCA4 gene. The cell lines are split based on expression loss of SMARCA1. The boxplot labeled 0 has SMARCA1 expression loss, and the boxplot labeled 1 has SMARCA1 normal expression. Cell lines appear more dependent on SMARCA4 when SMARCA1 is lost indicating synthetic lethality. Data is from the Hoffman epigenetic screen. **d** The biological rationale for synthetic lethality. SMARCA4 and SMARCA1 functionally redundant domains and gene ontology terms

SMARCA1 is a tumour suppressor gene whose function is to facilitate the perturbation of chromatin structure. SMARCA4 is an epigenetic transcriptional co-activator. Both are SNF2 chromatin remodeling ATPases [32] and there exists a large amount of functional redundancy between the pair (GO terms including chromatin remodeling and chromatin binding). SMARCA4 has also previously been described as synthetic lethal to another SNF2 chromatin remodeling ATPase – SMARCA2 [26]. The SMARCA1 / SMARCA4 genes are never expressed at low levels together (Fig. 5a, S = 6.49), and the integrated-genomic workflow revealed that mutation / loss of SMARCA4 occurs at much lower frequency where there are low levels of SMARCA1 expression (Fig. 5b, *p* = 0.12). Here we see that in two of the three ATARIS solutions for SMARCA4, the phenotype dependency score is significantly lower where there is a low expression level of SMARCA1 (Fig. 5c, *p* = 0.02).

#### SETD1A and TGFB1

SETD1A is a histone methyltransferase and important epigenetic target that forms part of the control mechanism for chromatin structure and gene expression [33]. TGFB1 is a growth factor with known linkage to histone methyltransferase targets [34]. Cell lines are significantly more dependent on SETD1A in the absence of TGFB1 at the gene expression level (Additional file 1: Figure S6, S = 4.36). Two SETD1A ATARIS solutions return a significant *p* value for this interaction (Additional file 3: Table S2, *p* = 0.006 & *p* = 0.01) and have a significantly lower ATARIS phenotype score.

#### SUV39H2 and NPDC1

SUV39H2 is a histone methyltransferase whose function is to generate a trimethylation tag for epigenetic transcriptional repression [35, 36]. NPDC1 is a transcriptional regulator of growth and proliferation in brain and lung tissue. In the absence of NPDC1 cell lines are significantly more dependent on SUV39H2 (Additional file 3: Table S2, S = 2.05), and two published SUV39H2 ATARIS solutions return a significant *p* value for this gene pair (Additional file 3: Table S5, *p* = 0.01 & *p* = 0.02). Loss of SUV39H2 could lead to changes in epigenetic silencing and cell differentiation, and a lower level of transcriptional repression leading to growth and proliferation of cells.

#### SETD1A and PRMT6

The methyltransferase SETD1A is also involved in a second compelling pairing with the epigenetic target gene PRMT6. PRMT6 is a methyltransferase that acts as a transcriptional repressor of genes such as TP53, and regulates base excision repair. Both genes are involved in the GO processes of chromatin re-organisation and assembly. This was the only gene pair where both members are epigenetic and present in both the CCLE cell line analysis (S = 1.49, Additional file 3: Table S2) and independently in the TCGA lung adenocarcinoma analysis (S = 2.63, Additional file 3: Table S9). Whilst overlap was insufficient between the cell lines used in our analysis and those used in the Hoffman screen to power the RNAi dependency test (52 of 54 available cell lines are in the high expression mode) (Additional file 1: Figure S7), it is nevertheless a compelling functionally redundant epigenetic pairing.

### In silico validation of nominated pairs using publically-available epigenetic shRNA screen data: integrated-genomic workflow

4,768 gene pairs from the integrated-genomic workflow included at least one epigenetic gene (Additional file 3: Table S4) and 41 pairs where both members were epigenetic genes.

#### CHD8 and FANCM

CHD8 is a DNA helicase that acts to suppress p53 mediated apoptosis as a transcription factor and a chromatin remodeling factor. FANCM is an ATPase required for the ubiquitination of FANCD2 and is important for repair of single strand breaks in DNA. The BEEM analysis data shows that where CHD8 mutation is completely mutually exclusive with loss of FANCM expression (Additional file 3: Table S1, *p* = 0.23). In the epigenetic RNAi screen, where FANCM expression is lost, cell lines show a greater dependency on CHD8 (*p* = 0.009).

#### NSD1 and KLHL9

**Fig. 6:**
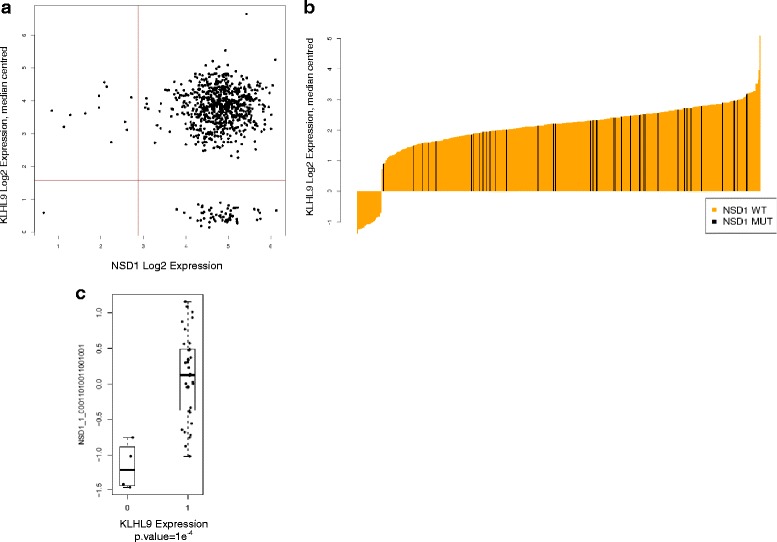
The KLHL9 and NSD1 synthetic lethal pairing. **a** The KLHL9 and NSD1 synthetic lethal pairing as seen by the gene expression workflow. The pair shows an almost complete mutually exclusive loss of expression across 811 cell lines (only the KS1 cell line prevents total mutually exclusive loss at the expression level). **b** The expression distribution of KLHL9 (orange) coloured by NSD1 mutation (black). This pair was detected by the genomic workflow (BEEM). NSD1 loss is exclusive where KLHL9 expression is high. This is indicative of possible interdependency. **c** The ATARIS phenotype dependency scores for cell lines on the NSD1 gene. The cell lines are split based on expression loss of KLHL9. The boxplot labeled 0 has KLHL9 expression loss, and the boxplot labeled 1 has SMARCA1 normal expression. Cell lines are more dependent on NSD1 when KLHL9 is lost indicating synthetic lethality. Data is from the Hoffman epigenetic screen

The highest scoring gene pair from the genomic workflow epigenetic analysis output (Fig. 6). NSD1 is a histone methyltransferase that acts as a transcriptional intermediary factor that regulates transcription [37]. KLHL9 is an adaptor of the BCR ubiquitin protein ligase complex that mediates the ubiquitination of AURKB. NSD1 is not expressed at low levels when KLHL9 is mutated (Additional file 3: Table S1, *p* = 0.15) and in the RNAi screen where NSD1 is expressed at low levels, cells are more dependent on KLHL9 (*p* = 9.48 × 10^−5^, Fig. 6).

## Discussion

Detection of synthetic lethalities between cancer tumor suppressors and potential drug targets offers great potential to advance therapeutic options in patients poorly served by therapies targeting activated oncogenes. To identify candidate synthetic lethal interactions, we considered inference of loss-of-function through low mRNA expression apparent in bimodal and non-normal gene expression distributions alongside, and as an alternative to, genetic loss of function. We have developed three workflows, presented as a collection of computational tools, to enrich for synthetic lethal gene-pair hypotheses through analysis of multi-omic datasets. Our approach focuses on the identification of synthetic lethal gene pairs, however readers should consider that further synthetic lethalities may exist involving larger collections of genes. We focus on mutual exclusivity of loss-of-function as a true measure of synthetic lethality, and assess pan-cancer data sets to avoid relationships contradicted in different tumor types.

Bimodality in gene expression may be driven by genetic deletion, activation or dependency present in a population of tumour cells. A heterogeneous tumour sample typical of a patient tumour biopsy, however, may comprise multiple cell populations. It is reasonable, therefore, to expect bimodality in such samples to be diluted and visible only as a non-normal distribution (Fig. 2). Non-normal gene expression distributions may also occur in homogeneous cell populations when you have high / low populations with vastly different sample size, as is often the case for tumour suppressor populations. BiSEp can accurately detect the mid-point of distributions between two (high and low expressing) populations regardless of whether it is manifested as clear bi-modality or as non-normality in shape. This enables genes with both characteristics to be included in the synthetic lethal analysis using the expression and mutation workflows.

Most prior attempts to predict synthetic lethalities in molecular data have focused on identifying mutually exclusive loss determined by genetic mutation or copy number alone. These approaches have shown some success identifying dependencies between genes in parallel pathways, or genes with similar or housekeeping functions, but miss many well validated examples of synthetic lethality where at least one gene in the pair does not show genetic loss in tumours. This can include the only clinically prosecuted synthetic lethal pairing where genetic loss of BRCA1/2 leads to sensitivity to PARP1/2 inhibition with Lynparza (Olaparib, AZD2281), since PARP1/2 are not reported to show genetic loss in tumours. We have shown that by comparing loss inferred from bimodal gene expression, either directly or to genetic loss (mutation and/or gene deletion), we can extend our ability to identify validated synthetic lethal gene pairs beyond comparisons of genetic data alone. We have supplemented our validation by highlighting the ability of the toolkit to identify the gold standard known synthetic lethal pairing between BRCA / PARP (Fig. 4), and show with statistical significance that we find enrichment of tumour suppressor gene pairs (Additional file 1: Figure S3). Furthermore, and considering the precedent for synthetic lethality impacting cancer metabolism [8], using a published archive of established cancer metabolism genes [28] we found significant enrichment evident in the output of our expression-only (*p* < 0.002) and integrated-genomic workflows (*p* < 3.51 × 10^−7^).

A lack of a sizeable number of clinically (or experimentally) validated true positive synthetic lethal gene pairs in each human cancer setting makes it difficult to obtain a true measure of specificity and sensitivity of the prediction methods. To address this we used several data sets to assess if our workflows had enriched for validated synthetic lethalities, including overlap with (a) a set of synthetic sick/lethal pairs originally identified in yeast [23], (b) two sets of candidate synthetic lethal gene pairs from human cancer settings [8, 9], (c) RNAi data from a genome wide epigenetic screen [26] and (d) a known set of tumour suppressor genes [27]. In yeast, large screens have been performed to inhibit large numbers of gene pairs systematically, providing a set of inferred synthetic lethal/sick relationships to compare to. This is not an ideal validation dataset due to the significant divergence of the yeast and human genomes, and a fair assumption would be that many of the associations that were relevant in yeast are likely to be at best, less meaningful in human cell populations. None-the-less, highly significant overlap with these datasets suggests that we are enriching for biologically validated synthetic lethal pairs (Fig. 3). To build on this finding we demonstrated a significant overlap of our results to candidate synthetic lethal genesets from Lu et al. [9] and Jerby-Arnon et al. [8] (Additional file 1: Figure S5), but also an important complimentarity where validated synthetic lethal gene pairs were uniquely found by each method alone.

The principal goal of our approach is to sufficiently enrich for synthetic lethal hypotheses to enable rational testing in loss-of-function shRNA/CRISPR screens. Although our methods reduce the search space for candidate synthetic lethality very significantly by 99.98 %, this still leaves tens of thousands of putative pairwise synthetic lethal interactions. Certain steps may be taken to further prioritize hypotheses for testing relevant to a particular research interest. For example analyses were run on pan-cancer cell line data to avoid relationships contradicted in different tumor types, however since a number of cancer pathway and gene’s function is limited to certain tumor types this may have introduced false positive results. To further prioritize relevant to a disease of interest it would be pertinent to focus on molecular relationships reproducible in patient samples from that disease, as we have demonstrated in lung cancer with the TCGA data. Alternatively hypotheses may be reduced to those aligned to a biological area of focus using the gene functional annotation provided, as demonstrated for DNA repair and epigenetics. Furthermore our characterization in tumor cell lines, and respective annotation, presents an opportunity to rationally design a functional-genomic screen by identifying the cell line with a specific molecular deficiency in gene 1 and the associated gene 2 pairings predicted synthetic lethal.

Candidates overlapping to dependencies found in human cancer cell RNAi data were further investigated for functional redundancy and a firm biological rationale. The clinical success seen exploiting synthetic lethalities to prosecute DNA repair deficiencies led us to study the enrichment of DNA repair genes in the outputs from the integrated-genomic and expression-only workflows. We identify marginal enrichment of these genes in our toolkit and discovery a compelling gene pairing from within the geneset; ERCC4 / XRCC1. There is some rationale for these targets as a synthetic lethal pairing as up-regulation of ERCC4 may enable XRCC1 deficient cells to maintain viability. Conversely cells deficient in ERCC4 may rely on up-regulation of XRCC1 in order to reduce the likelihood of replication fork stalls.

Identifying new therapeutic targets with a synthetic lethal hypothesis in the epigenetic space offers similar potential. Inhibiting various components of the epigenetic machinery may help restore normal chromatin function, which has been found to be affected by abnormalities in epigenetic genes in cancer. To address this we combined gene pairs nominated by the pure gene expression and expression/mutation workflows with RNAi data from the Hoffman screen. This approach has enabled us to validate gene pairs nominated by the *in silico* methods and to prioritise those that look more biologically compelling as candidate synthetic lethal genes. We have discovered compelling evidence for synthetic lethality between several different gene pairs involving epigenetic target genes. SMARCA1 / SMARCA4 are both ATP dependent helicase genes that are members of the SWI/SNF family of proteins and share a common functional domain structure (Fig. 5d). They both share a common function as transcriptional co-activators and the evidence the toolkit has highlighted at the molecular level is strong and backed up by the greater dependency seen on SMARCA4 in cell lines that have expression loss of SMARCA1 (Fig. 5a), indicative of a synthetic lethal interaction. In addition, SMARCA1 and SMARCA4 are one of a small number of gene pairs that overlap with interacting gene pairs retrieved from the BioGrid resource ([34], Additional file 1: Figure S3e). We have found evidence for two synthetic lethal interactions involving SETD1A – with TGFB1, and SETD1A and PRMT6. PRMT6 and SETD1A are both epigenetic methyltransferase genes with a direct functional redundancy, whereas TGFB1 is a well-known growth factor that has been linked to transcription of methyltransferase genes. The SETD1A / TGFB1 relationship is suggestive of redundancy during activation of epigenetic gene expression. The top hit from the genomic workflow output and epigenetic screen output is the pairing of NSD1 and KLHL9 (Fig. 6). The molecular and RNAi data are very compelling but there is little existing biological information to suggest why these two genes would be synthetic lethal. NSD1 has been implicated in cancers of the brain such as glioma and neuroblastoma [37] and there has been extensive work done on characterization of the NSD family including crystal structures for each of NSD1 / NSD2 and NSD3 [38]. KLHL9 has not been implicated in cancer, but highly homologous members of the KLHL family have such as KEAP1 and KLHL20 – both of which are notable tumour suppressors [39]. Despite the lack of evident biology for this pair, the relationship is so strong enough in the data that it should be considered for further validation. The other compelling pairing from the genomic workflow output is the pairing CHD8 and FANCM; both with links to DNA damage repair in the p53 signaling axis and a working hypothesis that if both genes are lost a cell may be pushed towards p53 mediated apoptosis, making inhibition of one where the other is lost an attractive therapeutic strategy.

## Conclusions

This work has been undertaken to provide a computational toolkit enriching for candidate signatures of dependency and synthetic lethality in large pan-molecular datasets, steering further validation towards the nomination of new targets for cancer therapy. Whilst there will be no substitute for gold-standard validation, it is hoped that this work can be a source of a reduced set of hypotheses testable through experiments using techniques such as siRNA or CRISPR. Notably the molecular nature of the relationship identified also facilitates identification of disease models and patients for this testing. We hope this work and these methods will ultimately lead to identification of new targets, contributing to therapies for patients with tumor suppressor driven cancers.

### Ethics approval

All samples and data used for these analyses were de-identified and publicly available, and where necessary were applied in accordance with ethical restrictions associated to access. No additional ethics approval was required for this study.

### Availability of data

The data sets supporting the results of this article are available in the Gene Expression Omnibus repository (GEO) [GSE36133], and the broad institute confluence portal [http://firebrowse.org].

## Additional files

Additional file 1: Figure S1. AUC and prediction gain scores for BISEP and several alternative algorithms. **Figure S2.** The effect of different balances of high / low expression distributions on the adjustment of the mid-point values in BiGEE. **Figure S3.** Tumour suppressor and DNA Repair enrichment and overlap in gene pairs nominated by the BISEP toolkit. **Figure S4.** Comparison of different SL enrichment methods using bootstrapping analysis. **Figure S5.** Candidate SL interaction between SETD1A and TGFB1. **Figure S6.** Candidate SL interaction between SETD1A and PRMT6. (PDF 748 kb) Additional file 2
**The RNAi analysis script, performed in R.** (R 39 kb) Additional file 3: Table S1. Output from the CCLE genomic workflow (BEEM) and mapping to genes from the Hoffman epigenetic screen, the Costanzo yeast screen and known tumour suppressor genes. **Table S2.** Output from the CCLE expression (BiGEE) workflow and mapping to genes from the Hoffman epigenetic screen, the Costanzo yeast screen and known tumour suppressor genes. **Table S3.** Output from the copy genomic (BEEM) workflow and mapping to genes from the Hoffman epigenetic screen, the Costanzo yeast screen and known tumour suppressor genes. **Table S4.** Epigenetic Analysis: Genomic workflow (BEEM). **Table S5.** Epigenetic Analysis: Expression workflow (BiGEE). **Table S6.** Output from the gene expression workflow (BiGEE) mapped to genes from the Lu et al. publication. **Table S7.** Output from the gene expression workflow (BEEM) mapped to genes from the Lu et al. publication. **Table S8.** Output from the LUAD Genomic (BEEM) workflow and mapping to genes from the Hoffman epigenetic screen, the Costanzo yeast screen and known tumour suppressor genes. **Table S9.** Output from the LUAD expression (BiGEE) workflow and mapping to genes from the Hoffman epigenetic screen, the Costanzo yeast screen and known tumour suppressor genes. **Table S10.** Simulation results for the likelihood method (*p* value), Kurtosis method (*p* value), bimodal index (1.1–1.5) and BIG (BiSEp) (*p* value). (XLSX 8250 kb)

## Abbreviations

BRCA: breast cancer susceptibility gene/protein
LUAD: lung adenocarcinoma
PARP: poly ADP ribose polymerase gene/protein
SL: synthetic lethality
TCGA: The Cancer Genome Atlas
