# Supplementary material for: Multi-omic measurement of mutually exclusive loss-of-function enriches for candidate synthetic lethal gene pairs
Source: BMC Genomics. 2016 Jan 19;17:65. doi: 10.1186/s12864-016-2375-1 (PMC4717622; doi:10.1186/s12864-016-2375-1)
Supplement: Additional file 1: Figure S1. — AUC and prediction gain scores for BISEP and several alternative algorithms. Figure S2. The effect of different balances of high / low expression distributions on the adjustment of the mid-point values in BiGEE. Figure S3. Tumour suppressor and DNA Repair enrichment and overlap in gene pairs nominated by the BISEP toolkit. Figure S4. Comparison of different SL enrichment methods using bootstrapping analysis. Figure S5. Candidate SL interaction between SETD1A and TGFB1. Figure S6. Candidate SL interaction between SETD1A and PRMT6. (PDF 748 kb) [file 12864_2016_2375_MOESM1_ESM.pdf]

Supplementary Material

1. The BiSEp algorithm

The BiSEp algorithm (Bimodality Subsetting Expression) pairs the bimodal index [1] with a novel approach BIG to score significance and clarity of bimodal and non-normal expression profiles, and identify the 'trough' or 'midpoint' threshold of this bimodality to dissect high from low expressing samples.

1.1 The novel component of BISEP: BIG

The focus of BIG is to accurately detect the midpoint of the bimodal distributed genes, including those whose bimodality may be diluted (as likely in a heterogeneous cell population) to a more non-normal profile. In order to do this we maximize consecutive differential expression (CDE) in a sorted expression list of a gene. The assumption is that the consecutive differential expressions follow a normal (Gaussian) distribution. Denote a set of CDEs by $\mathbf{x} = (x_1, x_2, \cdots, x_k)$, its mean by μ_k and its variance by σ_k^2. The relationship between x_{k+1} and the density $\mathcal{G}(\mu_k, \sigma_k^2)$ is $t_{k+1} = \frac{x_{k+1} - \mu_k}{\sigma_k}$. t_{k+1} can then be converted to a p value depending on the degree of freedoms. For a set of n CDEs, there will be n such p values. We select the minimum of the n p values, hence maximum CDE. Based on a critical p value setting, we can then make a decision whether the maximum CDE assigned to a gene is significantly deviated from the remaining CDEs. If so, the gene is predicted as bimodal. To calculate associated p values, we scan a set of CDEs sequentially and increase the elements of **x** incrementally. Starting from a minimum CDE set size for calculating mean and variance for all CDEs for a gene, the scan returns the p-value and the midpoint of the bimodal distribution.

The BISEP tool therefore combines the BI, π and δ values with the distribution midpoint value derived by BIG to detect both bimodal and non-normal expression profiles. This provides the user with more control over the shape of the bimodal distribution, and enables the dynamic partitioning of samples into high and low populations.

1.2 Comparing BISEP to other bimodality detection methods

We performed a comparison of the BIG component of BISEP with the likelihood approach [2], kurtosis approach [3] and bimodal index approach [1] detecting bimodality in continuous gene expression data (see simulated data analysis section below, simulations 1-6 are present in Supplementary Table 8). BISEP performs well in comparison to the other methods with high prediction gain and AUC values (probability the algorithm will rank randomly chosen gene higher than bimodal gene) comparable to the best performing 'likelihood' approach (Fig. S1).

1.2.1 Simulated data analysis

6 data scenarios were simulated:

Scenario 1 – Here we generated 1000 simulated genes. Of these genes, 950 were designed to be unimodal and 50 were designed to be bimodal. The sample number was 50. All unimodal genes and the 45 low expression samples were drawn from a random normal distribution with a mean of 10 and a standard deviation of 1 N (10, 1). The high expression samples of bimodal genes were drawn from a random normal distribution with a mean of 0 and a standard deviation of 1 N(0, 1) plus 2 above the maximum values of the low expression samples. Results plotted in Fig S1.

Scenario 2 – All the same as above except for the high expression samples which were drawn from a uniform distribution from zero to one plus two above the maximum value of the low expression samples.

Scenario 3 – Data size was the same as above but the distribution was different. Here, all samples of unimodal genes and the low expression samples of bimodal genes were drawn from a uniform distribution between 10 and 12. The high expression samples of the bimodal genes were drawn from a normal distribution from N (0, 0.5^2) above the maximum value of low expression samples.

Scenario 4 - Data size is still the same as above. All samples of unimodal genes were drawn from N (10,1). The low expression samples of bimodal genes were drawn from a mixture of N (10,1) and N (11,1). The high expression samples of bimodal genes were drawn from N (0,0.5^2) plus two above the maximum value of low expression samples.
Scenario 5. Data size is still the same as above. All samples of unimodal and the low expression samples of bimodal genes were drawn from N (10,1). The low expression samples were drawn from a mixture of N (10,3), N(11,2) and N(12,1). The high expression samples were drawn from a uniform distribution between zero and one plus two above the maximum low expression
Scenario 6. Data size is still the same as above. All samples of unimodal and the low expression samples of bimodal genes were drawn from N(10,1). The low expression samples were drawn from a mixture of N(10,3), N(11,2) and N(12,1). The high expression samples were drawn from a mixture of N(1,1) and N(2,1) plus two above the maximum low expression.

We used two measurements for the evaluation. The first one is AUC - area under ROC curve. The second measurement is called prediction gain, which is a ratio between sensitivity and false discovery proportion. Given a confusion matrix shown in table 1, the sensitivity is defined as W/(V+W) and the false discovery proportion is defined as Q/(Q+W).

Table 1

	unimodal	bimodal
Unimodal	F	Q
Bimodal	V	W

The full simulation results are available in supplementary table S10.

2. References

1. Wang, J, Wen S, Symmans WF, Pusztai L & Coombes K. 2009. The Bimodality Index: A criterion for Discovering and Ranking Bimodal Signatures from Cancer Gene Expression Profiling Data. Cancer Informatics 7, 199-216
2. Fraley, C and Raftery, AE. 2002. Model-based clustering, Discriminant Analysis and Density Estimation. Journal of the American Statistical Association, 97 (458) 611-630.
3. Klar B. 2002. A Treatment of Multivariate Skewness, Kurtosis, and Related Statistics. Journal of multivariate analysis 83: 141-165

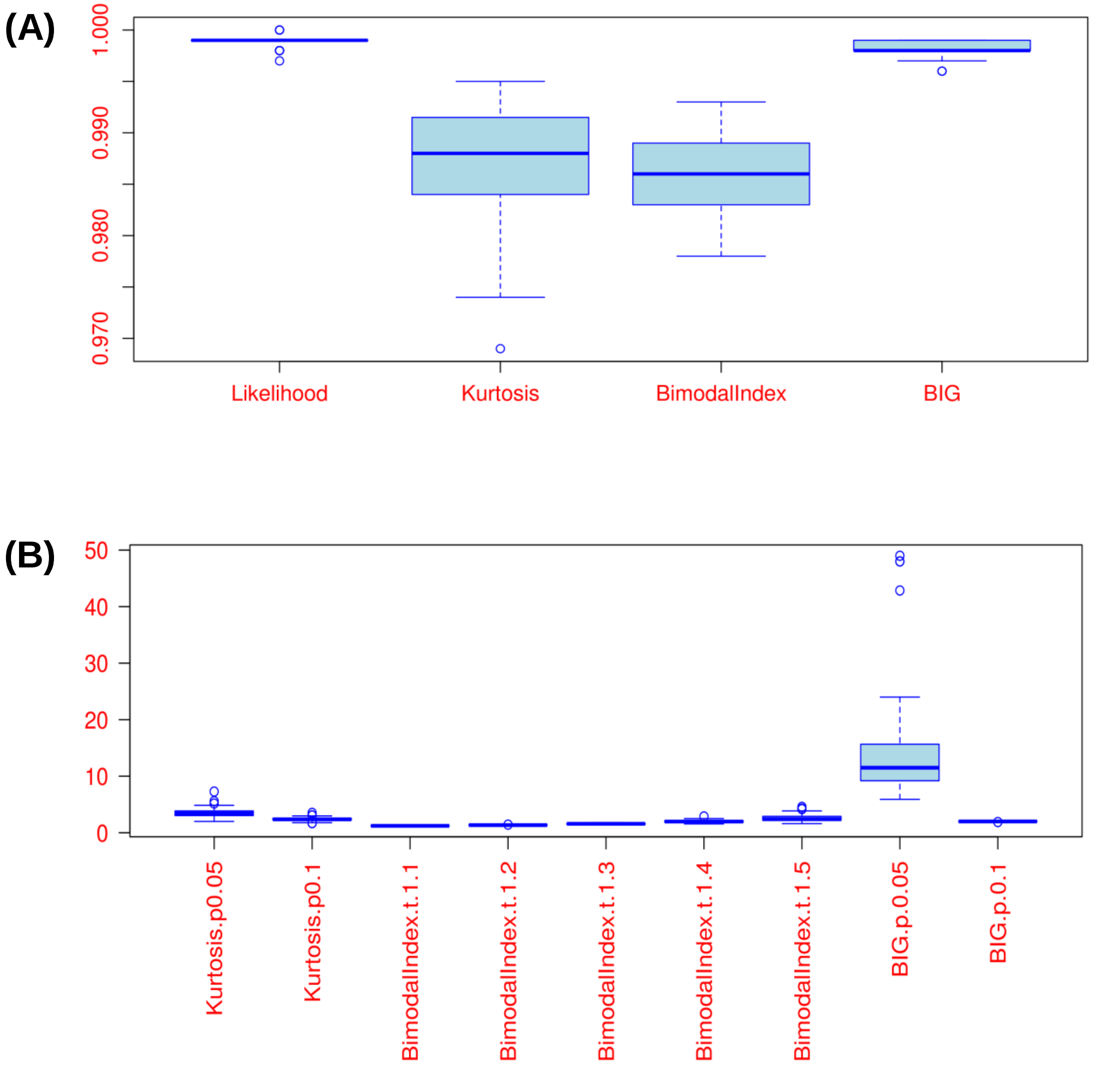

Figure S1
(A) AUC (area under ROC-curve) score for bimodality data simulation. A geneset of n = 1000 was simulated with 950 unimodal and 50 bimodal genes.

(B) Prediction gain score (ratio between sensitivity and false discovery proportion) for bimodality data simulation 1.

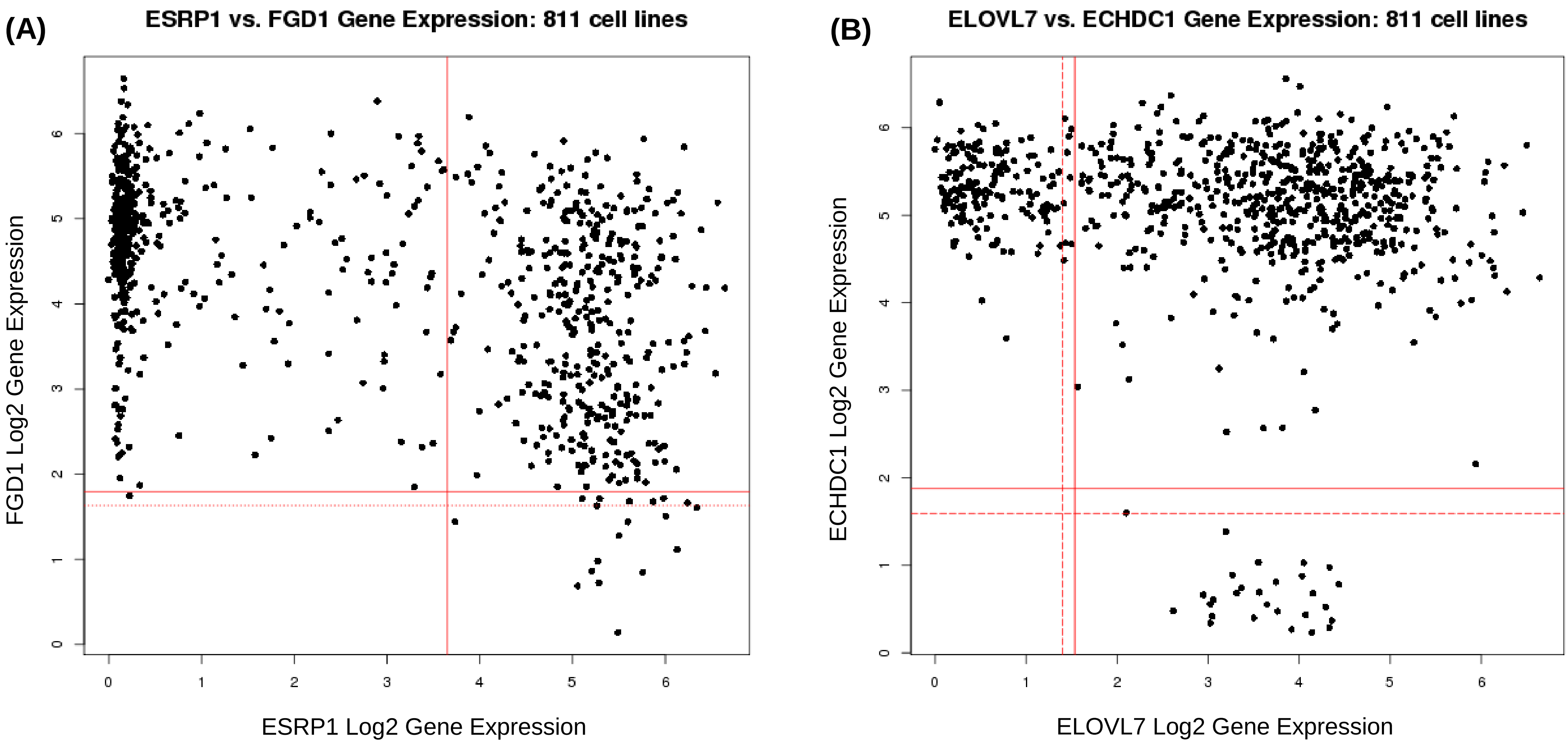

Figure S2

The effect of different balances of high / low expression distributions on the adjustment of the mid-point values in BIGEE. The lower the π value, the more unbalances the expression distribution is and the midpoint is adjusted to be a less rigid cut-off.

(A) FGD1 is adjusted by 2.5% of the total expression distribution as π value is 0.27. ESRP1 is adjusted by 0% of the total expression distribution as π value is 0.48.

(B) ELOVL7 is adjusted by 2% of the total expression distribution as π value is 0.30. ECHDC1 is adjusted by 4.5% of the total expression distribution as π value is 0.04

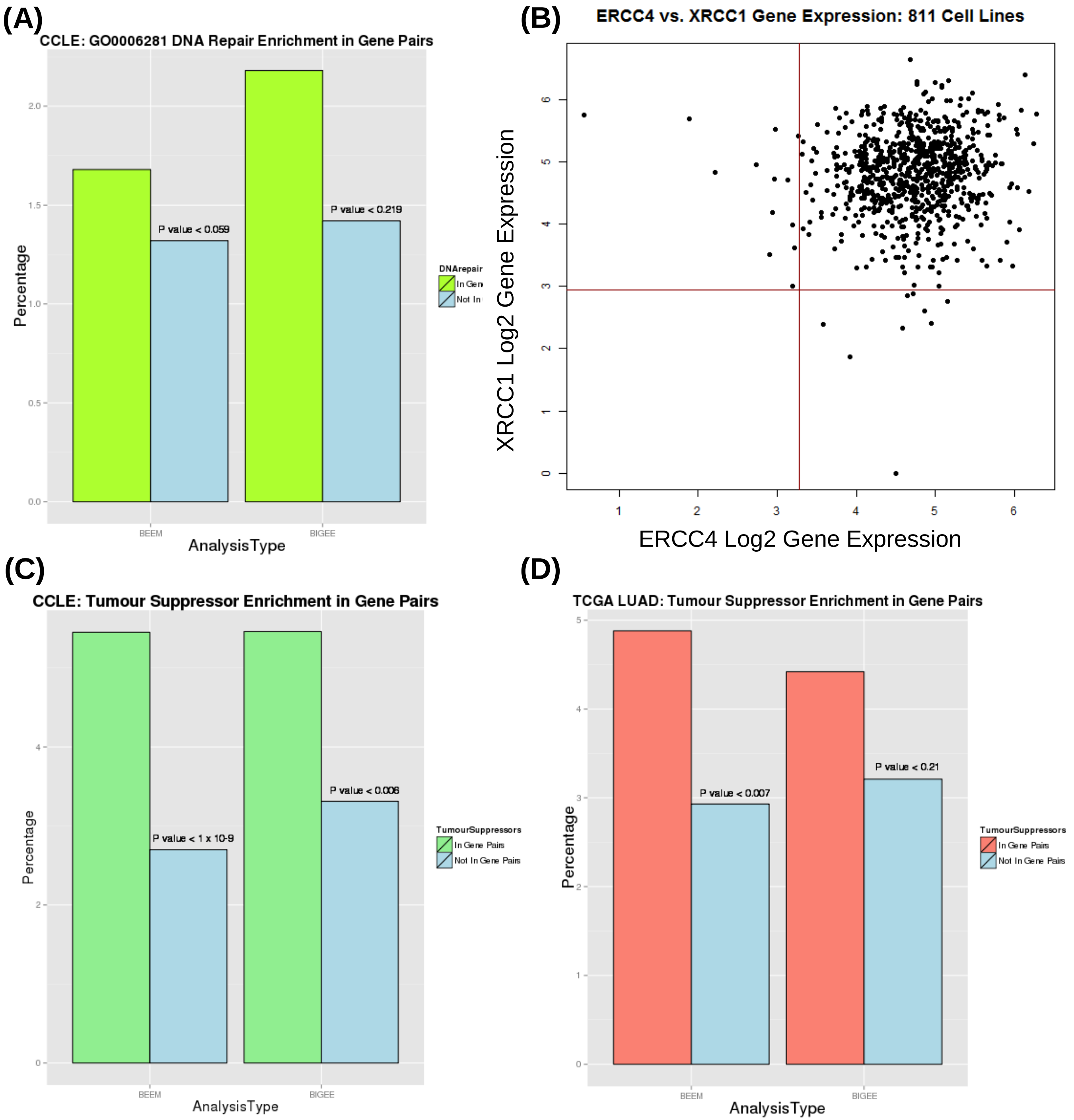

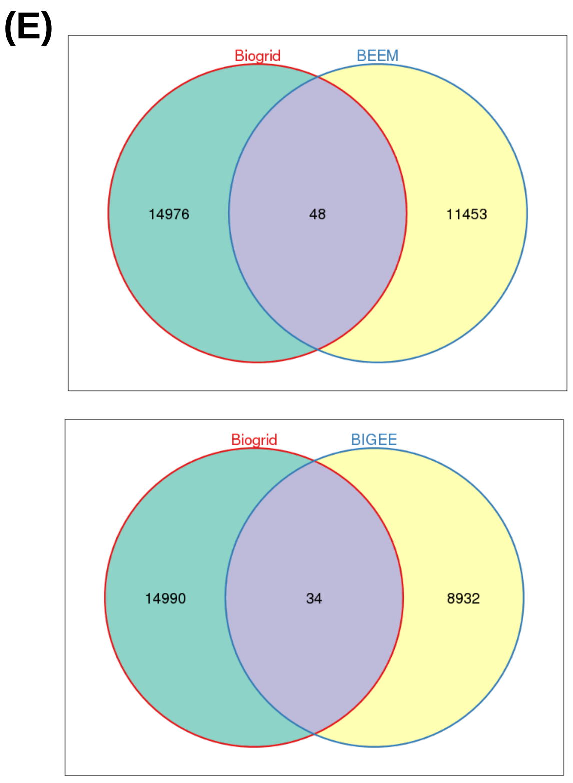

Figure S3

Tumour suppressor and DNA Repair enrichment and overlap in gene pairs nominated by the BISEP toolkit. Overlap between tumour suppressor interactions and output from the BioGrid protein interaction database.

(A) CCLE cell line DNA Repair overlap
(B) ERCC4 / XRCC1 candidate tumour suppressor pair
(C) CCLE cell line data tumour suppressor overlap.
(D) TCGA lung adenocarcinoma tumour suppressor overlap.
(E) Overlap between tumour suppressor pairs and the BioGrid protein interaction database.

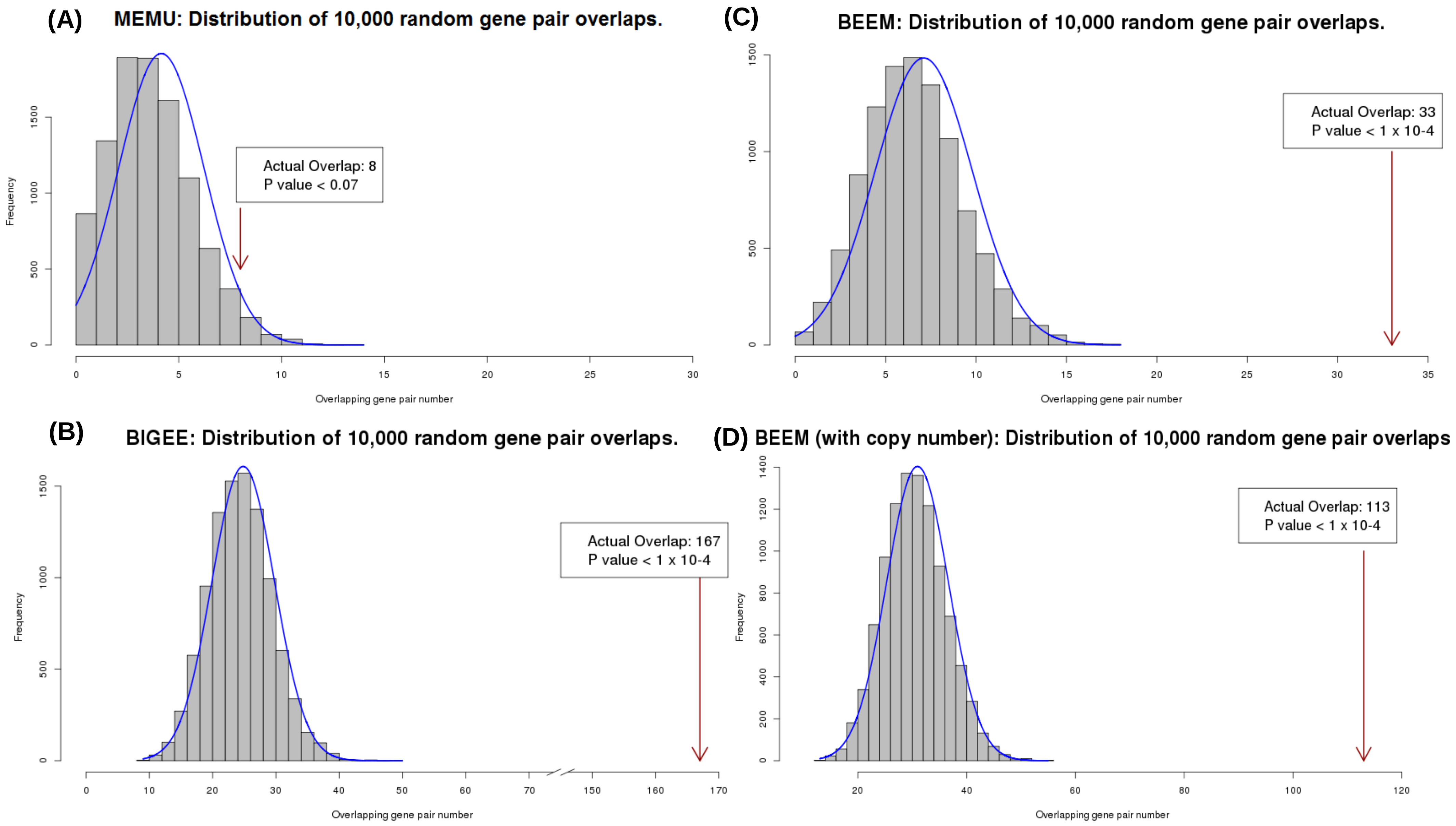

<u>Figure S4</u>.
(A)The gene pair outputs of mutually exclusive mutation (MEMU) analysis, (B) the expression workflow (BIGEE), (C) the genomic workflow (BEEM) and (D) the genomic workflow (BEEM) including discreet copy number calls, are overlapped with the human orthologues of synthetic lethal yeast gene pairs. This overlapped number is compared to 10,000 random samplings of the same size of the gene pair outputs to establish how the workflows perform when compared to chance.

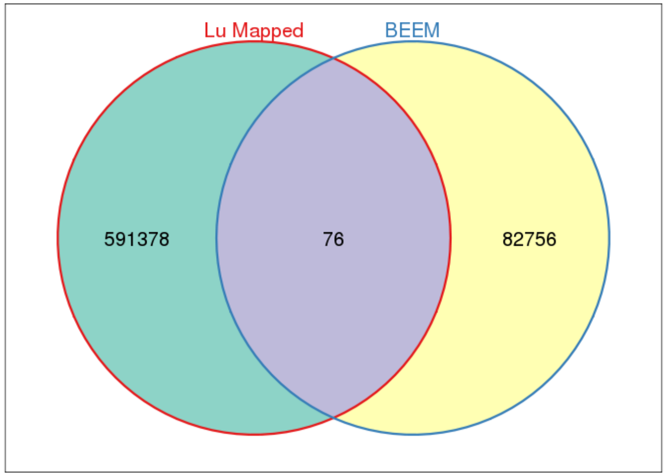

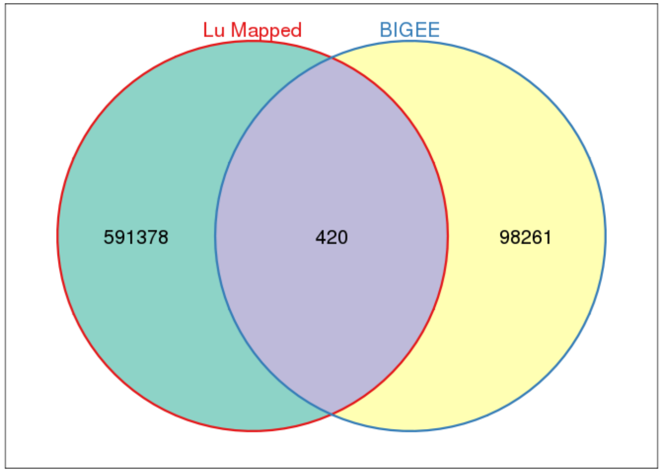

Figure S5
(A)The gene pair overlaps between (A) the integrated genomic workflow (BEEM) and (B) the expression workflow (BIGEE) are overlapped with the gene pair outputs of the Lu *et al* human synthetic lethal analysis. This overlapped number is compared to 10,000 random samplings of the same size of the gene pair outputs to establish how the workflows perform when compared to chance (C). The individual genesets from the genomic (BEEM) and expression (BIGEE) workflows are overlapped with the geneset from the DAISY publication (D).

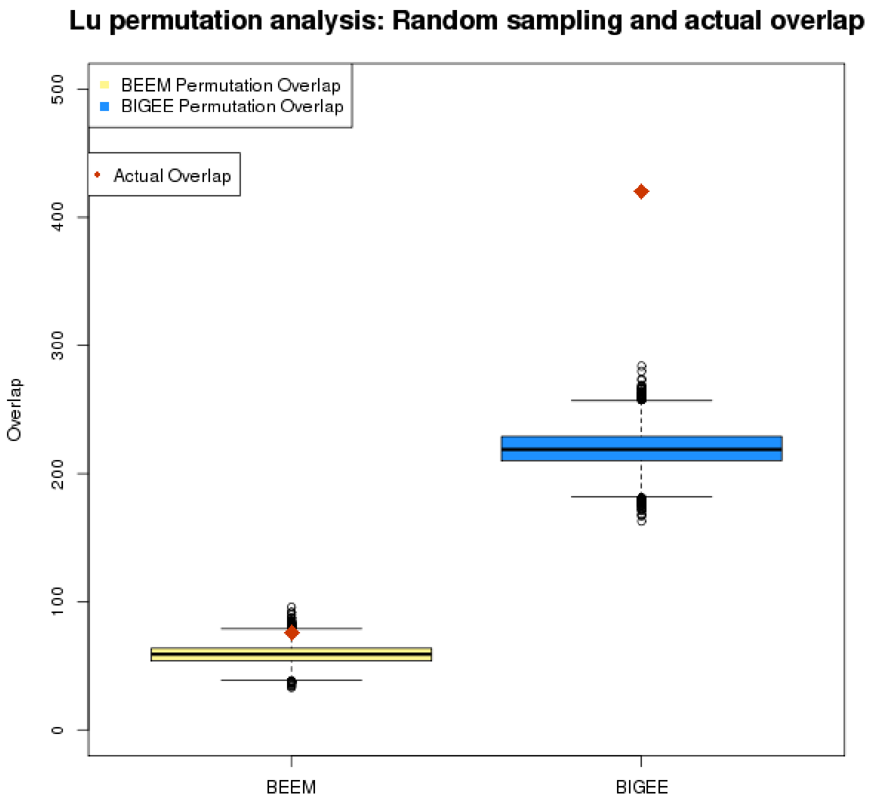

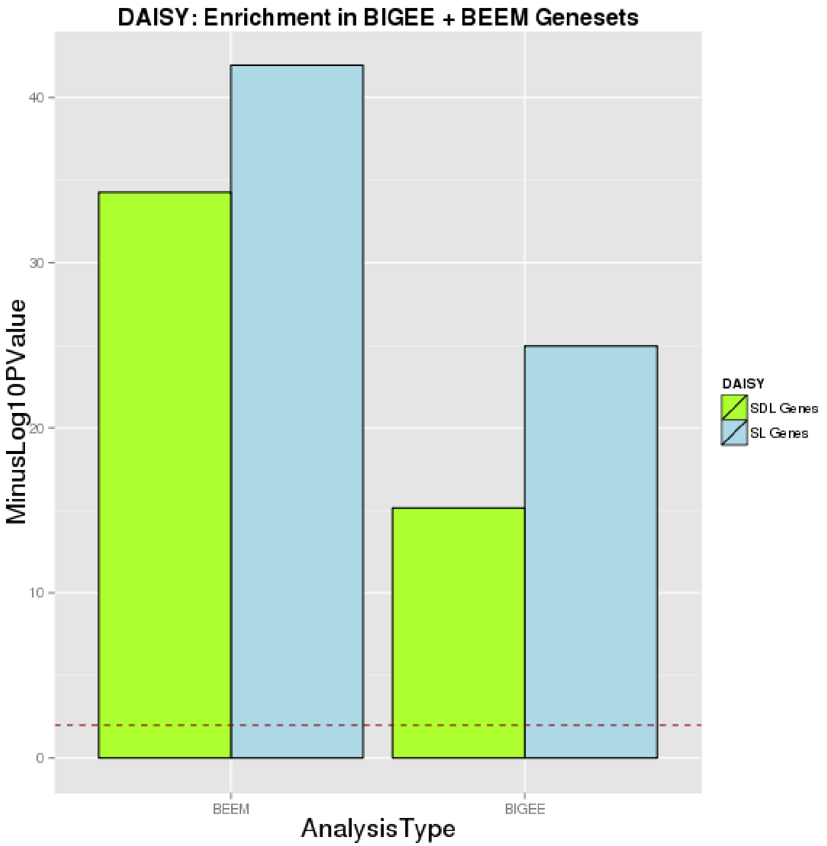

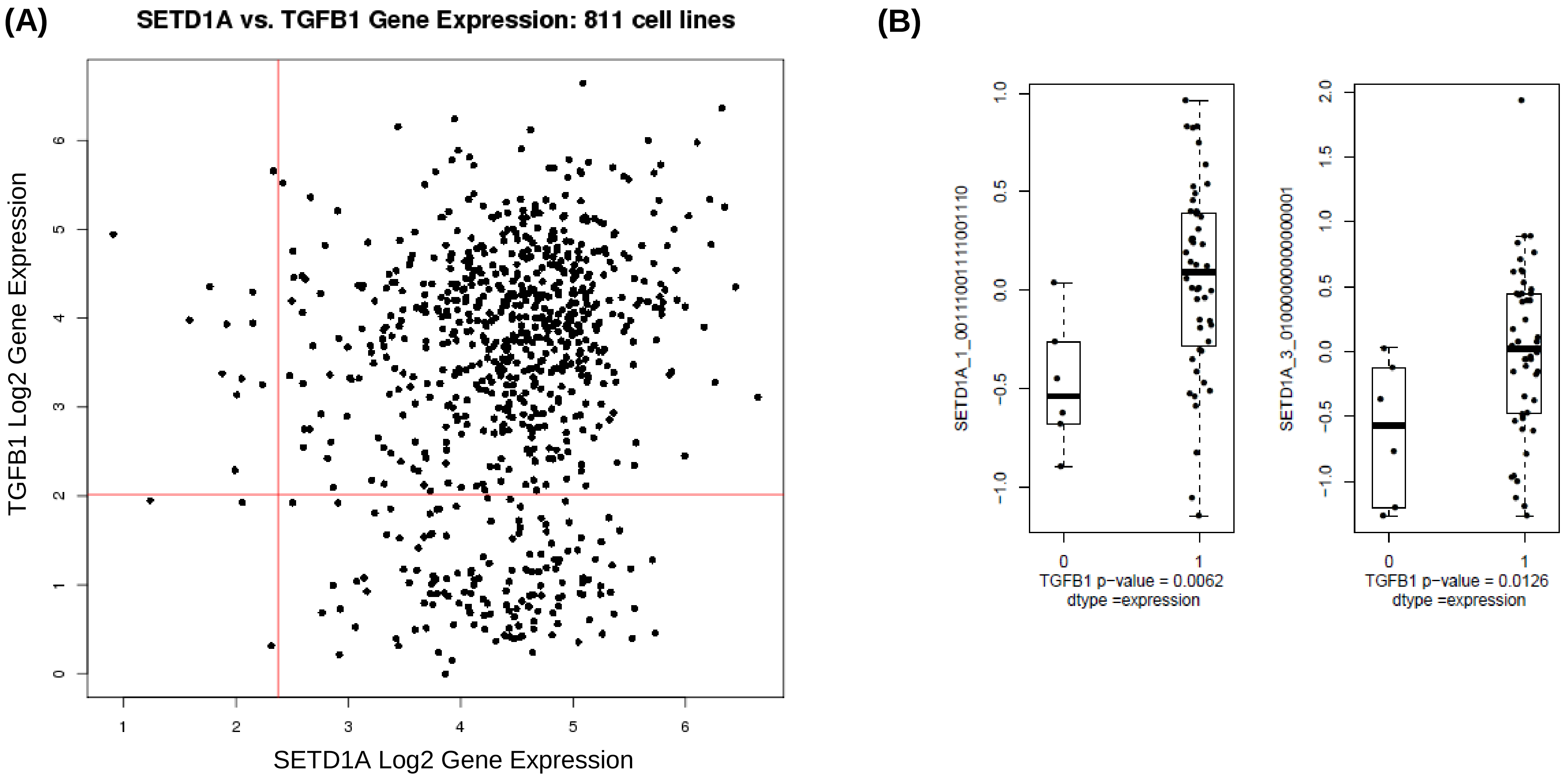

<u>Figure S6</u>

Candidate SL interaction between SETD1A and TGFB1

(A) Mutually exclusive loss of expression between the epigenetic target SETD1A and TGFB1

(B) Hoffman epigenetic screen score, BIGEE. SETD1A / TGFB1.

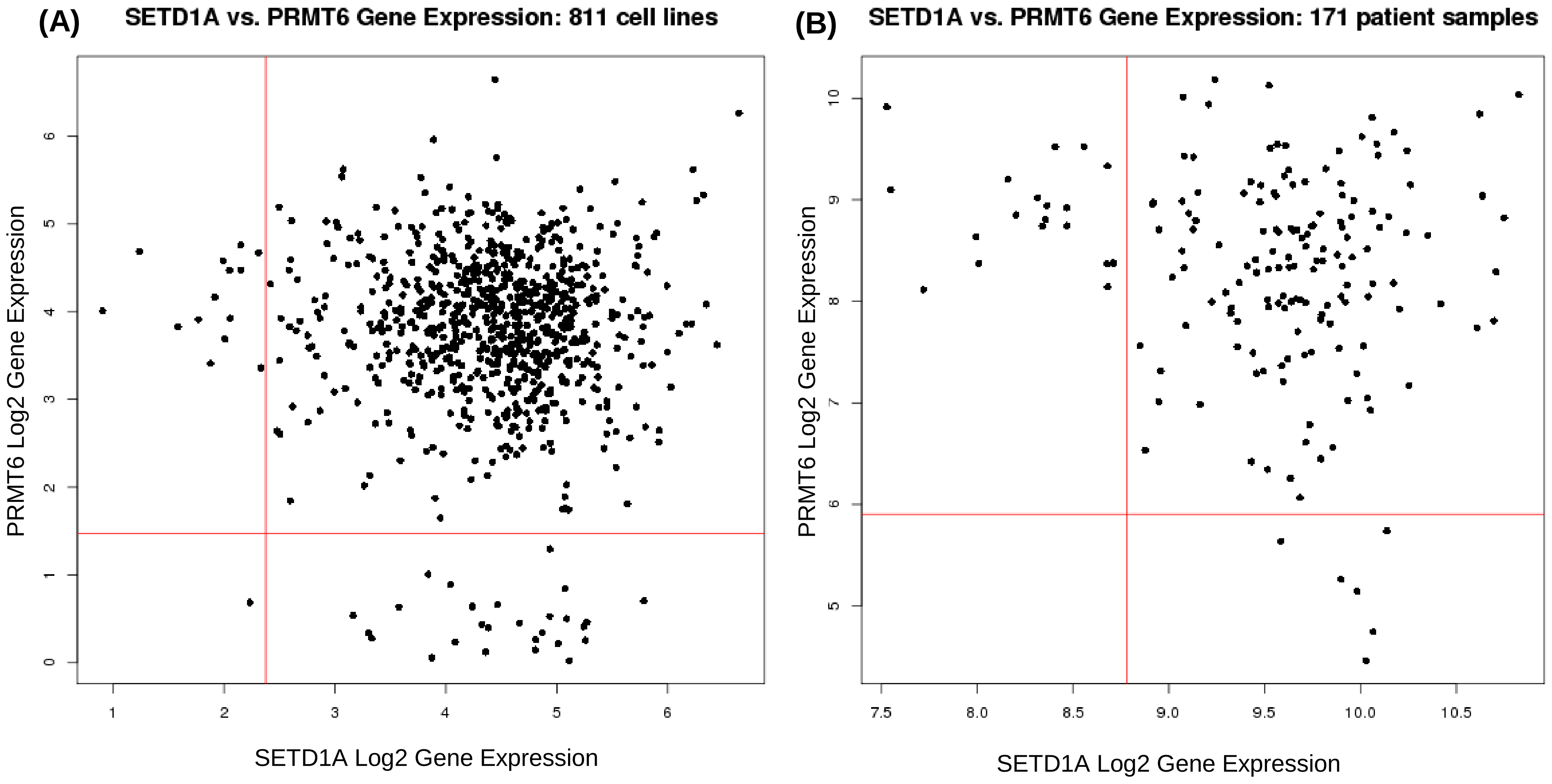

Figure S7

Candidate SL interaction between SETD1A and PRMT6

(A) Mutually exclusive loss of expression between the epigenetic target SETD1A and PRMT6: 811 CCLE cell lines.

(B) Mutually exclusive loss of expression between the epigenetic target SETD1A and PRMT6: 171 TCGA Lung adenocarcinoma samples.
